# Large Scale Functional Brain Networks Underlying Temporal Integration of Audio-Visual Speech Perception: An EEG Study

**DOI:** 10.3389/fpsyg.2016.01558

**Published:** 2016-10-13

**Authors:** G. Vinodh Kumar, Tamesh Halder, Amit K. Jaiswal, Abhishek Mukherjee, Dipanjan Roy, Arpan Banerjee

**Affiliations:** ^1^Cognitive Brain Lab, National Brain Research CentreGurgaon, India; ^2^Centre for Behavioural and Cognitive Sciences, University of AllahabadAllahabad, India

**Keywords:** EEG, AV, multisensory, perception, functional connectivity, coherence, temporal synchrony, integration

## Abstract

Observable lip movements of the speaker influence perception of auditory speech. A classical example of this influence is reported by listeners who perceive an illusory (cross-modal) speech sound (McGurk-effect) when presented with incongruent audio-visual (AV) speech stimuli. Recent neuroimaging studies of AV speech perception accentuate the role of frontal, parietal, and the integrative brain sites in the vicinity of the superior temporal sulcus (STS) for multisensory speech perception. However, if and how does the network across the whole brain participates during multisensory perception processing remains an open question. We posit that a large-scale functional connectivity among the neural population situated in distributed brain sites may provide valuable insights involved in processing and fusing of AV speech. Varying the psychophysical parameters in tandem with electroencephalogram (EEG) recordings, we exploited the trial-by-trial perceptual variability of incongruent audio-visual (AV) speech stimuli to identify the characteristics of the large-scale cortical network that facilitates multisensory perception during synchronous and asynchronous AV speech. We evaluated the spectral landscape of EEG signals during multisensory speech perception at varying AV lags. Functional connectivity dynamics for all sensor pairs was computed using the time-frequency global coherence, the vector sum of pairwise coherence changes over time. During synchronous AV speech, we observed enhanced global gamma-band coherence and decreased alpha and beta-band coherence underlying cross-modal (illusory) perception compared to unisensory perception around a temporal window of 300–600 ms following onset of stimuli. During asynchronous speech stimuli, a global broadband coherence was observed during cross-modal perception at earlier times along with pre-stimulus decreases of lower frequency power, e.g., alpha rhythms for positive AV lags and theta rhythms for negative AV lags. Thus, our study indicates that the temporal integration underlying multisensory speech perception requires to be understood in the framework of large-scale functional brain network mechanisms in addition to the established cortical loci of multisensory speech perception.

## Introduction

Perception of the external world involves the efficient integration of information over multiple sensory systems (Wallace et al., 1993). During speech perception, visual cues from the speaker's face enhances the intelligibility of auditory signal (Sumby, 1954; Helfer, 1997; Bulkin and Groh, 2006). Also, the incidence of specific *semantically-incongruent* visual information modulates auditory perception, for example, an auditory speech sound /*ba*/ superimposed with a speaker's lip movement of /*ga*/, gives rise to a perception of /*da*/ (McGurk and Macdonald, 1976). Similarly, an incongruent AV combination of /*pa*/-/*ka*/ elicits an ‘illusory’ (cross-modal) percept /*ta*/(McGurk and Macdonald, 1976; MacDonald and McGurk, 1978; van Wassenhove et al., 2007). However, such multisensory-mediated effects are influenced by the relative timing of the auditory and visual inputs (Stein et al., 1989; Munhall et al., 1996; Sekuler et al., 1997; van Atteveldt et al., 2007; van Wassenhove et al., 2007). Consequently, the temporal processing of the incoming multiple sensory (auditory and visual) information and their integration to yield a crossmodal percept is pivotal for speech perception (Deroy et al., 2014). Where and how the underlying information processing takes place is subject of several research studies which we review in the following paragraph. Cortical and sub-cortical regions and functional brain networks with specific patterns of connectivity becomes the prime target for these investigations. In a nutshell, characterization of the multi-scale representational space of temporal processing underlying multisensory stimuli is an open question to the community.

As we discuss in the following paragraph, a dominant strategy in multisensory research is the search for loci comprising of brain areas that are responsible for triggering the multisensory experience (Jones and Callan, 2003; Beauchamp, 2010; Nath and Beauchamp, 2013). However, from the perspective of functional integration (Bressler, 1995; Bressler and Menon, 2010) understanding the large-scale network organization underlying the temporal processes is a critical component of formulating a comprehensive theory of multisensory speech perception. Numerous neuroimaging and electrophysiological studies have explored the neural mechanism that underpins audio-visual integration employing McGurk effect (Wallace et al., 1993; Jones and Callan, 2003; Sekiyama et al., 2003; Kaiser, 2004; van Wassenhove et al., 2005; Hasson et al., 2007; Saint-Amour et al., 2007; Skipper et al., 2007; Stevenson et al., 2010; Keil et al., 2012; Nath and Beauchamp, 2013). A majority of these studies accentuate the role of primary auditory and visual cortices, multisensory areas such as posterior superior temporal sulcus (pSTS) (Jones and Callan, 2003; Sekiyama et al., 2003; Nath and Beauchamp, 2011, 2013) and other brain regions including frontal and parietal areas (Callan et al., 2003; Skipper et al., 2007) in the perception of the illusion. In particular, the electrophysiological evidences primarily emphasizes the significance of beta (Keil et al., 2012; Roa Romero et al., 2015) and gamma band activity (Kaiser, 2004) toward illusory (cross-modal) perceptual experience. Source-level functional connectivity among brain areas employing phase synchrony measures, reveal interactions among cortical regions of interest (left Superior Temporal Gyrus) and the whole brain that correlates with cross-modal perception (Keil et al., 2012). These studies either reveal the activations in the cortical loci or the functional connectedness to particular cortical regions of interest that are elemental for the illusory percept. On the other hand, the role of timing between auditory and visual components in AV speech stimuli has been studied from the perspective of the main modules in multisensory processing (Jones and Callan, 2003). Recently, we have addressed this issue using a dynamical systems model to study the interactive effects between AV lags and underlying neural connectivity onto perception (Thakur et al., 2016). Interestingly, how these network are functionally connected in the context of behavioral performance or perceptual experience are increasingly being revealed (Nath and Beauchamp, 2011; Keil et al., 2012). Nonetheless, the identification and systematic characterization of these networks under cross-modal and unimodal perception is an open question.

A traditional measure of large-scale functional connectivity in EEG is the sensor-level global coherence (Cimenser et al., 2011; Balazs et al., 2015; Fonseca et al., 2015; Alba et al., 2016; Clarke et al., 2016). Global coherence can be described as either the normalized vector sum of all pairwise coherences between sensor combinations, the frequency domain representation of cross-correlation between two time-series (Lachaux et al., 1999; Cimenser et al., 2011) or the ratio of the largest eigenvalue of the cross-spectral matrix to the sum of its eigenvalues (Mitra and Bokil, 2008). An increased global coherence confirms the presence of a spatially extended network that spans over several EEG sensors, since local pairwise coherence would not survive statistical threshold after averaging. To the best of our knowledge, global coherence has not been used in the domain of audio-visual (AV) speech perception to evaluate the presence of whole brain networks. Furthermore, characterization of the differences in whole brain network organization underlying cross-modal vs. unimodal perceptual experience vis-à-vis the timing of sensory signals will be critical to understanding the neurobiology of multisensory perception.

In the current study, we used the incongruent McGurk pair (audio /*pa*/ superimposed on the video of the face articulating /*ka*/) to induce the illusory percept /*ta*/. Further, we generated a temporal asynchrony in the onset of audio and visual events of the McGurk pair to diminish the rate of cross-modal responses. Subsequently, we exploited the inter-trial perceptual variability to study integration both at behavioral levels by accounting perceptual response and eye-tracking as well as neural levels using EEG. We considered subjects' /*pa*/ responses as unimodal perception since it represents only one sensory stream and /*ta*/ responses as cross-modal perception since it represents an experience resulting from integrating features from two modalities (Deroy et al., 2014). We studied the spectral landscape of perceptual categorization as function of AV timing and found patterns that matched with previous reports. Finally, we evaluate the large-scale brain network organization dynamics using time-frequency global coherence analysis for studying perceptual categorization underlying different temporal processing scenarios at various AV lags. In the process, we reveal the complex spectro-temporal organization of networks underlying multisensory perception.

## Materials and methods

### Participants

Nineteen [10 males and 9 females, ranging from 22–29, (mean age 25; *SD* = 2)] healthy volunteers participated in the study. No participant had neurological or audiological problems. They all had normal or corrected-to-normal vision and were right handed. The study was carried out following the ethical guidelines and prior approval of Institutional Review Board of National Brain Research Centre, India.

### Stimuli and trials

**Figure 1:**
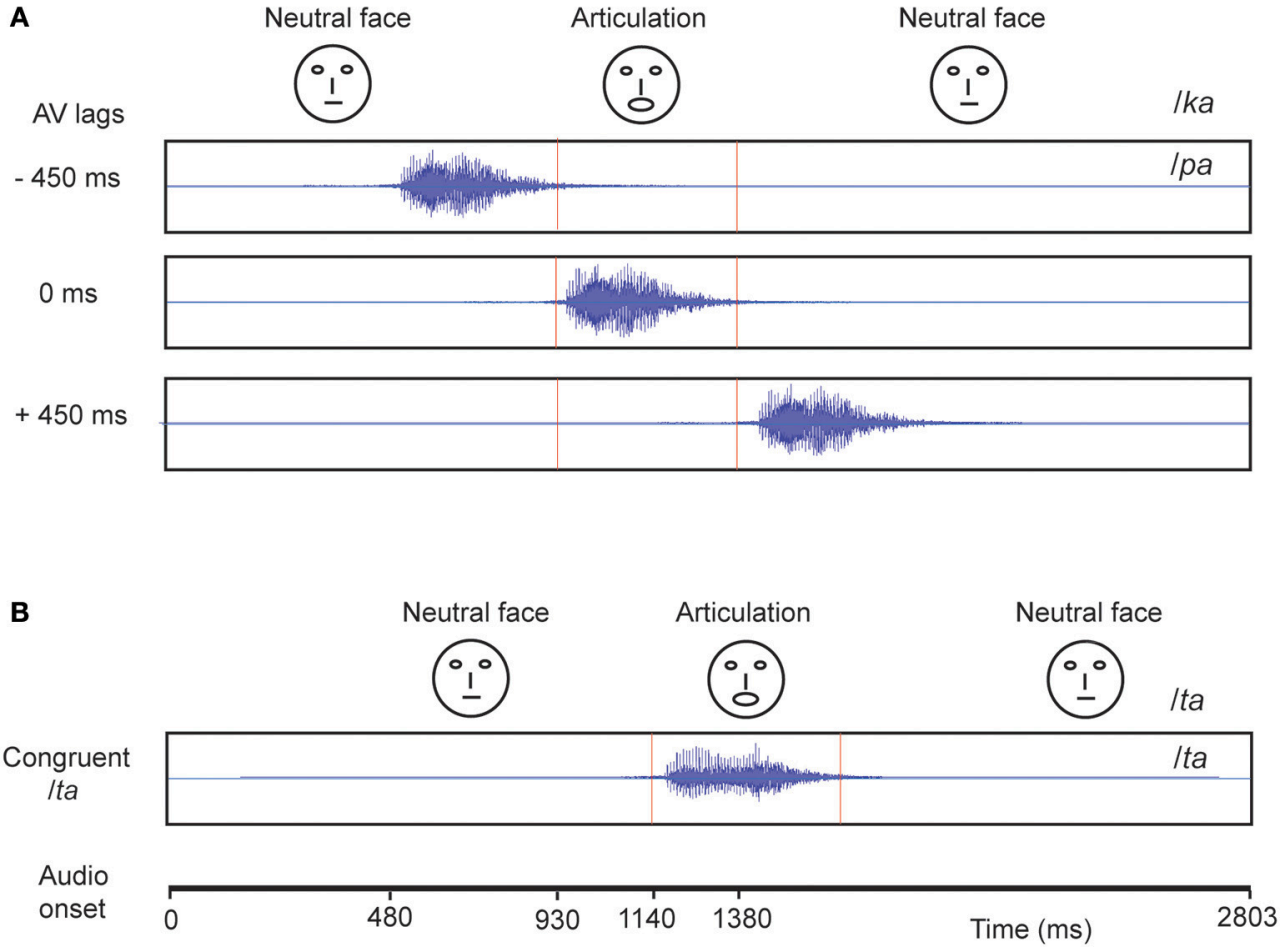
**Stimuli Each block represents a video**. **(A)** The McGurk stimuli: Audio */pa/* superimposed on visual (lip movement) */ka/* was presented under different audio-visual (AV) lag scenarios. The location of onset of audio is varied with respect to a person's initiation of lip-movement */ka/* at −450, 0, and 450 ms. **(B)** In congruent */ta/* condition, audio */ta/* is presented synchronously with onset of lip movement */ta/*.

The experiment consisted of 360 trials overall in which we showed the videos of a male actor pronouncing the syllables /*ta*/ and /*ka*/ (Figure 1). One-fourth of the trials consisted of congruent video (visual /*ta*/ auditory /*ta*/) and the remaining trials comprised incongruent videos (visual /*ka*/ auditory /*pa*/) presented in three audio-visual lags: −450 ms (audio lead), 0 ms (synchronous), +450 ms (audio lag), each comprising one-fourth of the overall trials. The stimuli were rendered into a 800 × 600 pixels movie with a digitization rate of 29.97 frames per second. Stereo soundtracks were digitized at 48 kHz with 32 bit resolution. The stimuli were presented via Presentation software (Neurobehavioral System Inc.). The video was presented using a 17″ LED monitor. Sounds were delivered at an overall intensity of ~60 dB through sound tubes.

The experiment was carried out in three blocks each block consisting of 120 trials. Inter-trial intervals were pseudo-randomly varied between 1200 and 2800 ms. Each block comprised the four stimuli types (30 trials of each): Congruent video and three incongruent videos with the AV lags. The subjects were instructed to report what they heard while watching the articulator using a set of three keys. The three choices were /*pa*/, /*ta*/ and “anything else” (Other).

Post EEG scan, the participants further performed a behavioral task. The task comprised of 60 trials, comprising 30 trials each of auditory syllables /*pa*/ and /*ta*/. Participants were instructed to report their perception using a set of two keys while listening to syllables. The choices were /*pa*/ and /*ta*/.

### Data acquisition and analysis

#### EEG

EEG recordings were obtained using a Neuroscan system (Compumedics NeuroScan, SynAmps2) with 64 Ag/AgCl sintered electrodes mounted on an elastic cap of Neuroscan in a 10–20 montage. Data were acquired continuously in AC mode (sampling rate, 1 kHz). Reference electrodes were linked mastoids, grounded to AFz. Channel impedances were kept at < 5 kΩ. All subsequent analysis was performed in adherence to guidelines set by Keil et al. (2014).

#### Eye tracking

**Figure 2:**
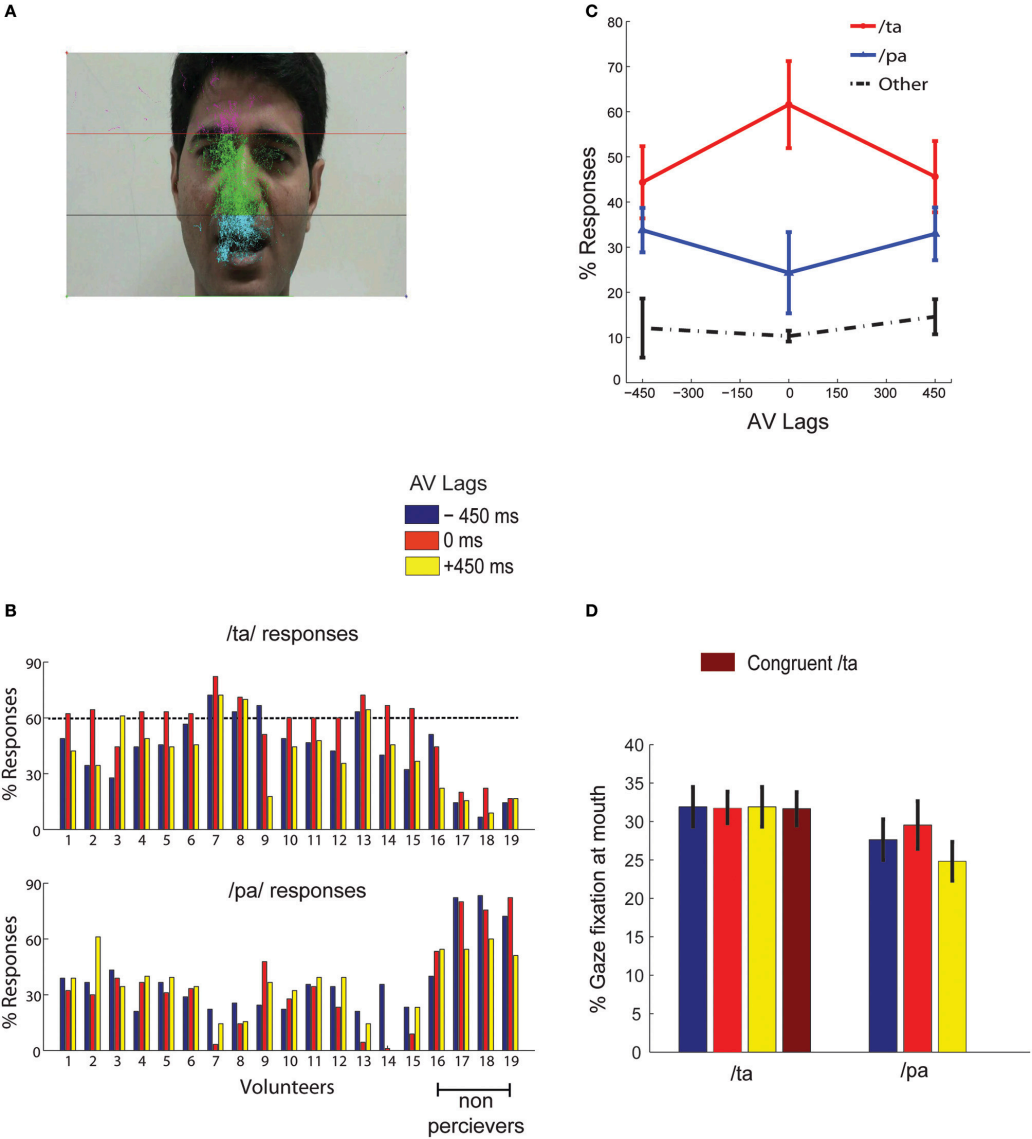
**Behavior (A) overall eye gaze fixation overlaid over a single frame of the stimuli (B) the bar graphs show the percentage of /***ta***/ and /***pa***/ responses for each subject at the AV lags:−450, 0, +450 ms as indicated by the colors guide (C) shows the number of normalized group responses in each of the three perceptual categories: “***/pa/***”, “***/ta/***”, and “other” for each AV lag**. The error bars represent the 95% confidence interval **(D)** Mean gaze fixation percentages at mouth for each perceptual category at the respective stimuli (incongruent AV lags −450, 0, +450 ms, and congruent /*ta*/) across trials and participants. The error bars represents 95% confidence interval. /*pa*/ perception for congruent /*ta*/ stimulus were less than < 1%.

Gaze fixations of participants on the computer screen were recorded by EyeTribe eye tracking camera with resolution 30 Hz (https://theeyetribe.com/). The gaze data were analyzed using customized MATLAB codes. The image frame of the speaker video was divided into 3 parts, the head, the nose and the mouth (Figure 2A). The gaze locations at these quadrants over the duration of stimulus presentation were converted into percentage measures for further statistical analysis.

#### Pre-processing of EEG signals

The collected EEG data were subsequently filtered using a bandpass of 0.2–45 Hz. Epochs of 400 and 900 ms before and after the onset of first stimuli (sound or articulation) were extracted and sorted based on the responses, /*ta*/, /*pa*/, and “other” respectively. Epochs were baseline corrected by removing the temporal mean of the EEG signal on an epoch-by-epoch basis. Epochs with maximum signal amplitude above 100 μV or minimum below −100 μV were removed from all the electrodes to eliminate the response contamination from ocular and muscle-related activities. Approximately 70–75 % (~250 trials) trials of each subject were preserved after artifact rejection. In the final data analysis, a mean of 24 (*SD* = 9), 18 (*SD* = 9), and 25 (*SD* = 13) incongruent trials at −450, 0, +450 ms AV lags respectively in which the participants responded /*pa*/ were included. Similarly, a mean of 32 (*SD* = 15), 42 (*SD* = 13), and 32 (*SD* = 14) incongruent trials at −450, 0, +450 ms AV lags respectively in which the participants responded /*ta*/ were included in the final analyses. Approximately 2–6% of trials were excluded from each of the aforementioned trial categories. The response category with lowest number of occurrences was */pa/* at 0 ms AV lag with 270 hits from a total of 1350 trials across all volunteers (15 × 90). Subsequently, we randomly resampled 270 trials from */ta/* responses at 0 ms AV lag, and */pa/* and */ta/* responses at other AV lags. Thus, for each AV lag condition, 270 trials chosen randomly from the respective sorted response epochs (*/pa/* or */ta/*) entered the final analyses.

#### Spectral analysis

Power spectra of the preprocessed EEG signals at each electrode were computed on a single trial basis. We computed the spectral power at different frequencies using customized MATLAB (www.mathworks.com) codes and the Chronux toolbox (www.chronux.org). Time bandwidth product and number of tapers were set at 3 and 5 respectively while using the Chronux function mtspecgramc.m to compute the power spectrum of the sorted time series in EEG data. Subsequently, the differences in the power during /*ta*/ and /*pa*/ responses at each AV lag were statistically compared by means of a cluster-based permutation test (Maris and Oostenveld, 2007) using the fieldtrip toolbox (www.fieldtriptoolbox.org). The fieldtrip function ft_freqstatistics.m was used to perform the cluster computation. During the statistical comparison, an observed test statistic value below the threshold of 0.05 in at least 2 of the neighborhood channels were set for being considered in the cluster computation. Furthermore, 1000 iterations of trial randomization were carried out for generating the permutation distribution at a frequency band. Subsequently, a two tailed test with a threshold of 0.025 was used for evaluating the sensors that exhibit significant difference in power. Statistical analysis was carried out separately for alpha (8–12 Hz), beta (13–30 Hz), and gamma (30–45 Hz) frequency ranges.

#### Large-scale network analysis

For deciphering the coordinated oscillatory brain network underlying the AV integration, we employed global coherence analyses (Bressler et al., 1993; Lachaux et al., 1999; Maris et al., 2007; Cimenser et al., 2011) on the perceptual categories (/*ta*/ and /*pa*/). A higher value of this measure will indicate the presence of strong large-scale functional networks. We computed the global coherence by decomposing information from the cross-spectral matrix employing the eigenvalue method (Mitra and Bokil, 2008). The cross-spectrum value at a frequency *f* between sensor pair *i* and *j* was computed as:

(1) $$\begin{array}{l} C_{ij}^{X}\left(f\right)=\frac{1}{K}\overset{K}{\underset{k=1}{\sum }}X_{i}^{k}\left(f\right)X_{j}^{k}{\left(f\right)}^{*} \end{array}$$

where $X_{i}^{k}$ and $X_{j}^{k}$ are tapered Fourier transforms of the time series from the sensors *i* and *j* respectively, at the frequency *f*. A 62 × 62 matrix of cross spectra, that represents all pairwise sensor combination, was computed in our case. Conversely, to characterize the dynamics of coordinated activity over time, we evaluated the time-frequency global coherogram. We employed the Chronux function cohgramc.m to obtain the time-frequency cross-spectral matrix for all the sensor combinations. Subsequently, for each trial we obtained the global coherence at each time point and frequency bin by computing the ratio of the largest eigenvalue of the cross-spectral matrix to the sum of the eigenvalues employing the following equation:

(2) $$\begin{array}{l} C_{Global}\left(f\right)=\frac{S_{1}^{Y}\left(f\right)}{\overset{n}{\underset{i=1}{\sum }}S_{i}^{Y}\left(f\right)} \end{array}$$

where C_*Global*_(*f*) is the global coherence, $S_{1}^{Y}\left(f\right)$ is the largest eigenvalue and the denominator $\sum_{i=1}^{n}S_{i}^{Y}(f)$ represents the sum of eigenvalues of the cross-spectral matrix (Cimenser et al., 2011). Time-frequency global coherogram computed for /*ta*/ and /*pa*/ responses were further compared at each time point for significant difference in different frequency bands (alpha, beta, and gamma) by means of cluster-based permutation test (Maris et al., 2007).

For every frequency bin at each time point, the coherence difference between /*ta*/ and /*pa*/ was evaluated using the Fisher's *Z* transformation

(3) $$\begin{array}{l} Z\left(f\right)=\frac{\tanh^{-1}\left(C_{1}\left(f\right)\right)-\tanh^{-1}\left(C_{2}\left(f\right)\right)-\left(\frac{1}{2m_{1}-2}-\frac{1}{2m_{2}-2}\right)}{\sqrt{\frac{1}{2m_{1}-2}+\frac{1}{2m_{2}-2}}} \end{array}$$

where 2*m*_1_, 2*m*_2_ = degrees of freedom; *Z*(*f*)≈*N*(0, 1) a unit normal distribution; and *C*_1_ and *C*_2_ are the coherences at frequency *f*.

The coherence *Z*-statistic matrix obtained from the above computation formed the observed Z-statistics. Subsequently, from the distribution of observed *Z*-statistics, 5th and the 95th quantile values were chosen as upper and lower threshold i.e., the values below and above the threshold values respectively were considered in the cluster computation. Based on spectral adjacency (4–7 Hz, theta; 8–12 Hz, alpha; 13–30 Hz, beta; 30–45 Hz, gamma), clusters were selected at each time point. Consequently, cluster-level statistics were computed by taking the sum of positive and negative values within a cluster separately. Following the computation of the cluster-level statistics of the observed *Z*-statistics, 1000 iterations of trial randomization were carried out. For every iteration, cluster-level statistic was computed on the randomized trials to generate the permutation distribution. Subsequently, the values of observed cluster-level statistics were compared with the 2.5th and the 97.5th quantile values of the respective permutation distribution. The observed cluster-level statistics value that were below 2.5th and above 97.5th quantile consequently for two time points formed the negative and positive clusters respectively.

## Results

### Behavior

Behavioral responses corresponding to McGurk stimuli with the AV lags were converted to percentage measures for each perceptual category (/pa/, /ta/, or “other”) from all subjects. We set a minimum threshold of 60% of /ta/ response in any AV lag, −450, 0, and +450 ms to qualify a participant as an illusory perceiver. 15 participants passed this threshold and 4 participants failed to perceive above the set threshold (see Figure 2B). Data from only 15 perceivers were used for further group level analysis. We observed that maximum percentage of illusory (/ta/) responses occurred at 0 ms AV lag when the lip movement of the speaker was synchronous with the onset of auditory stimulus (Figure 2C). Also, the percentage of /*pa*/ responses was minimum at 0 ms AV lag. We ran one-way ANOVAs on the percentage responses for /*pa*/, /*ta*/, and “other” with AV lags as the variable. We observed that AV lags influenced the percentage of /*ta*/ [*F*_(2, 44)_ = 27.68, *p* < 0.0001] and /*pa*/ [*F*_(2, 44)_ = 5.89, *p* = 0.0056] responses. However, there was no influence of AV lags on “other” responses [*F*_(2, 44)_ = 0.36, *p* = 0.700]. We also performed paired Student's *t*-test on the percentage of responses (/*ta*/ and /*pa*/) at each AV lag. Insignificant differences of 10.20–11.40% were observed between /*ta*/ and /*pa*/ responses at −450 ms AV lag [*t*_(14)_ = 0.63, *p* = 0.27] and +450 ms AV lag [*t*_(14)_ = 0.45, *p* = 0.67] respectively. However, at 0 ms AV lag we observed the percentage of /*ta*/ responses were significantly higher by 36.58% than the percentage of /*pa*/ responses, *t*_(14)_ = 10.20, *p* < 0.0001. Furthermore, the hit rate of /*ta*/ responses during congruent /*ta*/ was observed to be 0.97. Also, the hit rate of /*ta*/ and /*pa*/ during auditory alone conditions were observed to be 0.96 and 0.98 respectively.

Gaze fixations at different locations on the speaker's, head, nose and mouth areas were converted into percentage measures trial-by-trial for each subject and stimuli conditions. Figure 2A indicates that most of the gaze fixations were around head, nose, and mouth areas only. We ran a repeated measures 2-way ANOVA on mean gaze fixation percentages across trials at mouth areas with lags and perceived objects (/*pa*/ or /*ta*/) as variables. No significant differences were found for gaze fixations across lags [*F*_(2, 89)_ = 0, *p* = 0.95] and perceptual categorization [*F*_(1, 89)_ = 1.33, *p* = 0.27) as well as their interactions [*F*_(2, 89)_ = 0.01, *p* = 0.85]. Number of /pa/ responses for congruent /ta/ stimulus was negligible (< 1%), to do meaningful statistical comparisons. We also performed paired Student's *t*-tests on the mean gaze fixation percentages for /*pa*/ and /*ta*/ responses at each lag. Increases in gaze fixation at mouth during /ta/ perception by 15.5 % at −450 ms AV lag [*t*_(14)_ = 0.90, *p* = 0.38], 7.2 % at 0 ms AV lag [*t*_(14)_ = 0.90, *p* = 0.38] and 28.54% at +450 ms AV lag [*t*_(14)_ = −0.32, *p* = 0.74] (see Figure 2D for the mean values) were not statistically significant.

### Oscillatory activity

**Figure 3:**
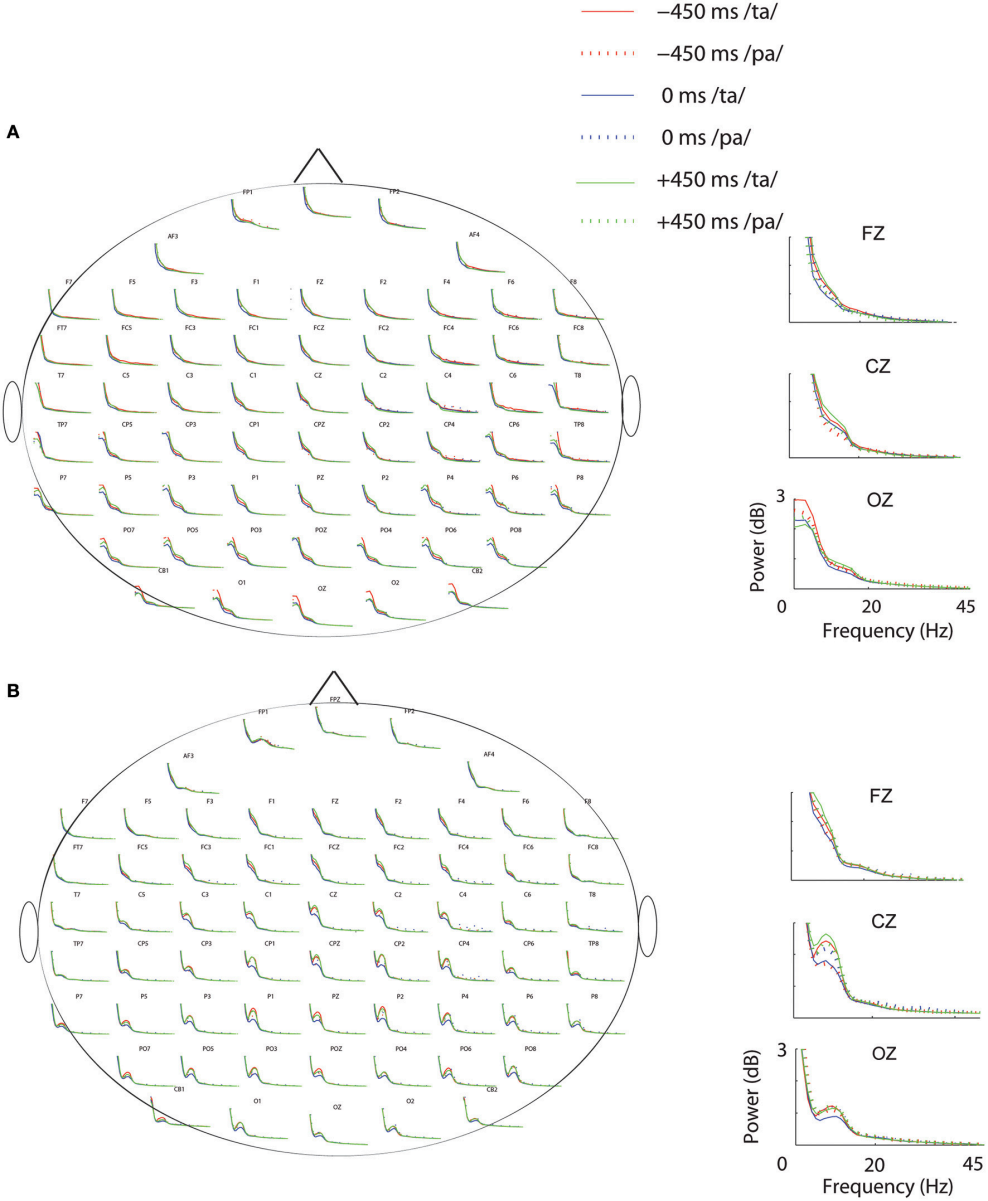
**Power Spectrum**. Spectral-power at each condition and perceptual category during **(A)** Pre-stimulus onset. **(B)** Post-stimulus onset periods. The plots adjacent to the scalp maps show the enlarged plots of the power spectrum at the sensors: Fz, Cz, and Oz.

**Figure 4:**
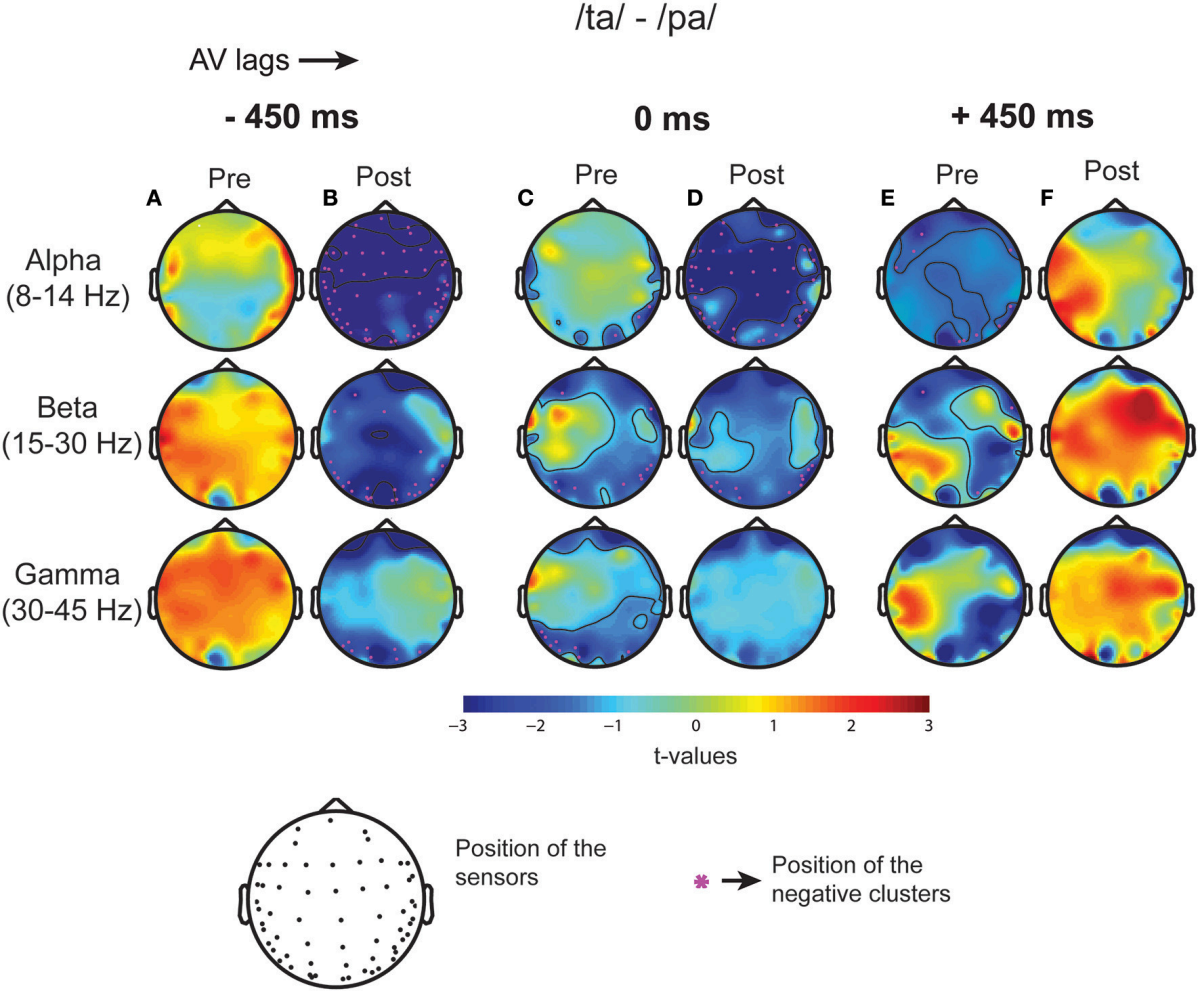
**Spectral Difference**. The topoplots and the magenta “*” highlight the clusters that show significant difference between the perceptual categories /*ta*/-/*pa*/ during the three stimulus conditions: −450 ms AV lag at **(A)** pre-stimulus onset **(B)** post-stimulus onset, 0 ms AV lag **(C)** pre-stimulus onset **(D)** post-stimulus onset, +450 ms AV lag **(E)** pre-stimulus onset **(F)** post-stimulus onset.

Subsequent to replicating the perceptual (Munhall et al., 1996; van Wassenhove et al., 2007) and the eye gaze behavior (Gurler et al., 2015) results as reported earlier, the focus of interest was what differentiates the two perceptual states (/*ta*/ and /pa/) in terms of brain oscillations and large-scale functional brain networks. Therefore, spectral power at different frequency bands during /*ta*/ and /pa/ perception were compared at different AV lags. Power spectra at each sensor computed in the time window before (see Figure 3A) and after (see Figure 3B) the onset of first stimuli showed distinct changes in power for the two states. Cluster-based permutation tests employed for comparing the spectral power between the perceptual states show that /*ta*/ perception is associated with an overall suppression in power for all AV lags (see Figure 4). The magenta “*” on the topoplots highlight the position of the negative clusters showing a significant suppression at 95% confidence levels in power. The blue areas on the scalp map highlight the regions that show decrease in the spectral power and the orange and red regions highlight the regions that show an increase in the spectral power. During the pre-stimulus period, one significant negative cluster [*t*_(269)_ = −2.04, *p* = 0.02] over temporo-occipital sensors, two over frontal and occipital sensors [*t*_(269)_ = −3.57, *p* = 0.002 and *t*_(269)_ = −3.14, *p* = 0.0002] and one over occipital sensors [*t*_(269)_ = −2.18, *p* = 0.01] were observed for alpha, beta, and gamma bands respectively in 0 ms AV lag (see Figure 4A). Also, one significant negative cluster over fronto-temporal and occipital sensors [*t*_(269)_ = −2.65, *p* = 0.004], one over frontal and occipital sensors [*t*_(269)_ = −2.31, *p* = 0.01] were observed at alpha and beta bands respectively during +450 ms AV lag (see Figure 4C). However, no significant difference was found during −450 ms AV lag.

Furthermore, during post-stimulus onset period, the /ta/-/pa/ comparison revealed one significant negative cluster over all sensors [*t*_(269)_ = −1.93, *p* = 0.02], one over frontal, parietal, and occipital sensors [*t*_(269)_ = −2.70, *p* = 0.004] and one over occipital sensors [*t*_(269)_ = −2.54, *p* = 0.006] at alpha, beta, and gamma bands respectively during −450 ms AV lag (see Figure 4C). During 0 ms AV lag, one significant negative cluster [*t*_(269)_ = −2.22, *p* = 0.01] spanning over all sensors and one over occipital sensors [*t*_(269)_ = −2.10, *p* = 0.02] was observed at alpha and beta bands respectively (see Figure 4D). However, no significant difference in power between /ta/-/pa/ trials was observed during the post-stimulus period at +450 ms AV lag. Overall, significant spectral power was lower during /ta/ than /pa/ as reflected from cluster-based analysis during pre- and post-stimulus periods.

### Time-frequency global coherogram

**Figure 5:**
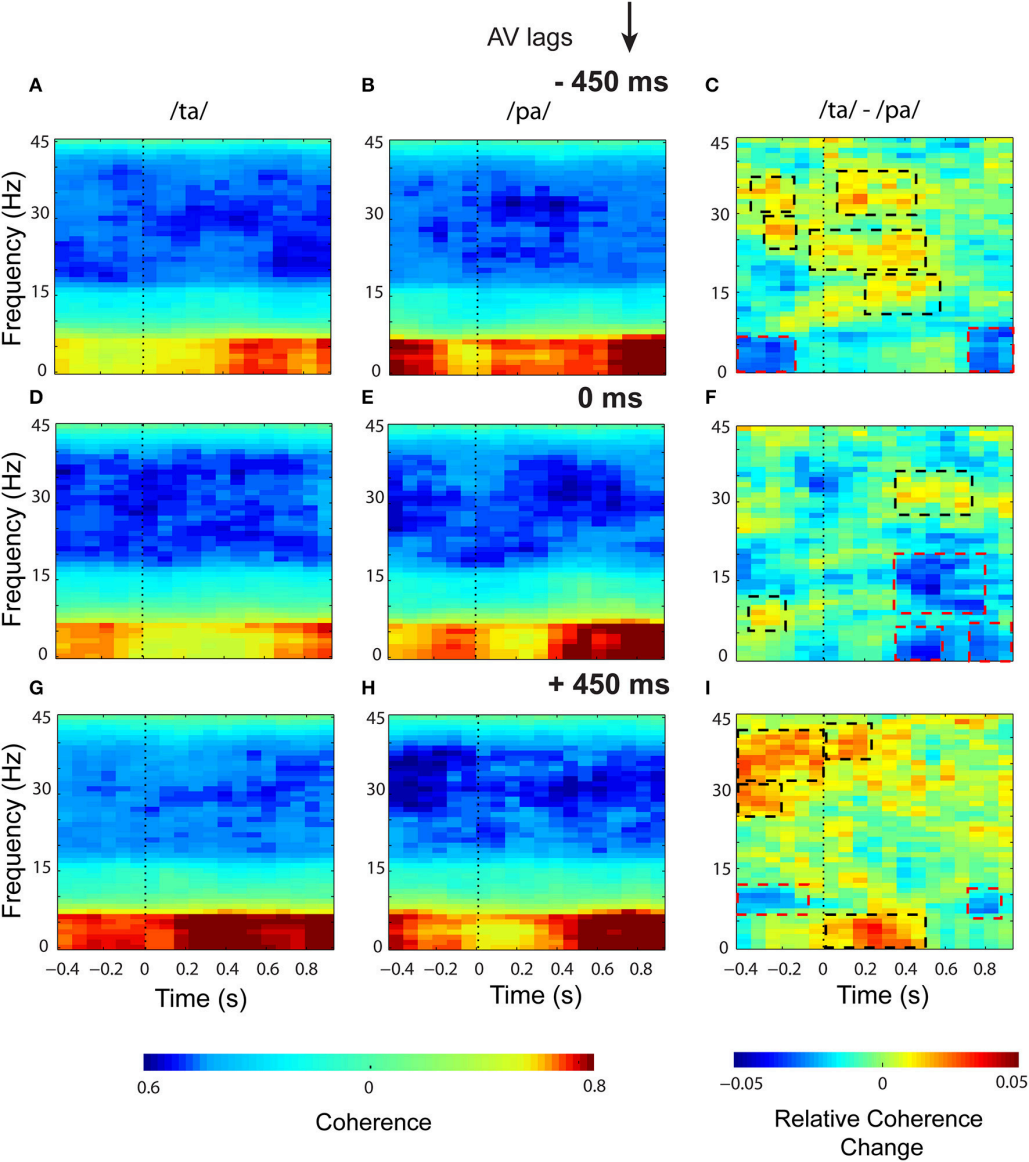
**Time-frequency representations of large-scale functional brain networks**. Mean time frequency coherogram for different perceptual categories time locked to the onset of the first sensory component (A or V) during the three conditions and the mean coherence difference between /*ta*/ and /*pa*/ responses at different AV lags: for −450 ms **(A)** /*ta*/ **(B)** /*pa*/ **(C)** /*ta*/-//*pa*/; for 0 ms **(D)** /*ta*/ **(E)** /*pa*/ **(F)** /*ta*/-//*pa*/; for 450 ms **(G)** /*ta*/ **(H)** /*pa*/ **(I)** /*ta*/-//*pa*/.

Eigenvalue based time-frequency global coherogram (Cimenser et al., 2011) was computed for the epochs of 1.3 s duration (0.4 s pre-stimulus, and 0.9 s post-stimulus segments). The time locking was done to the first sensory component, audio or visual, for −450 and +450 ms AV lag and the onset of AV stimulus for 0 ms AV lag. The mean coherogram plots for the perceptual categories /*ta*/ and /*pa*/ and their difference at AV lags: −450 ms (see Figures 5A–C), 0 ms (see Figures 5D–F), +450 ms (see Figures 5G–I) showed relatively heightened global coherence in the theta band (4–8 Hz) throughout the entire epoch duration. Cluster-based permutation tests employed to compare the mean coherogram for /*ta*/ and /*pa*/ at the respective AV lags revealed both positive and negative clusters (see Figures 5C,F,I). Positive clusters highlighted in black dashed rectangles signify time-frequency islands of increased synchrony and the negative clusters in red dashed boxes signify islands of decreased synchrony in the global neuronal network.

In the pre-stimulus period, we observed two positive and one negative cluster each during −450 and +450 ms AV lag. The first and second positive clusters during −450 ms AV lag were observed in the frequency bands beta (16–30 Hz) (*z*_97.5_ = 0.29) and gamma (>30 Hz) (*z*_97.5 = 0.78_) respectively and the negative cluster was found in theta band (4–7 Hz) (*z*_0.025_ = −0.29). Here, *z*_97.5_ and *z*_0.025_ represent the two-tailed thresholds at *p* = 0.05 set by permutation tests to compute the significantly different cluster (for details, see Methods section and Maris et al., 2007). Similarly during +450 ms AV lag the first and second positive clusters were observed in the frequency bands beta (*z*_97.5_ = 0.26) and gamma (*z*_97.5_ = 0.34) respectively and the negative cluster was found in the alpha band (8–12 Hz) (*z*_0.025_ = −0.78). However, during 0 ms AV lag, only a significant positive cluster was observed in the alpha frequency band (*z*_97.5_ = 0.58).

In the post-stimulus onset period, during −450 ms AV lag (see Figure 5C), three positive clusters were observed, (1) in alpha band with temporal range between ~200 and 560 ms (*z*_97.5_ = 0.50), (2) in beta band with temporal range between ~ −50 and 500 ms (*z*_97.5_ = 0.29), and (3) in gamma band between ~50 and 400 ms (*z*_97.5_ = 0.78). Also, a negative cluster (*z*_0.025_ = −1.02) was observed in the theta band between ~800 and 900 ms. During +450 ms AV lag (see Figure 5I), two positive clusters were observed, one in the theta band (*z*_97.5_ = 0.73) between ~0 and 500 ms and the other one in gamma band (*z*_97.5_ = 0.34) between ~ 0 and 200 ms. A negative cluster was also observed in the theta band (*z*_0.025_ = −0.68) between ~700 and 850 ms. Interestingly, during 0 ms AV lag (see Figure 5F) we observed a positive cluster (*z*_97.5_ = 0.26) precisely in the gamma band (~300 and 700 ms) and three negative clusters (*p* ≤ 0.05). Two of the negative clusters (*z*_0.025_ = 0.31) were observed in the theta band around 300 and 600 ms and ~700 and 900 ms and the third negative cluster incorporated both alpha and beta bands (9–21 Hz) (*z*_0.025_ = −0.25) and appeared between 300 and 800 ms.

## Discussion

Characterizing the dynamics of the whole brain network is essential for understanding the neurophysiology of multisensory speech perception. We have shown that the spatiotemporal dynamics of the brain during speech perception can be represented in terms of brain oscillations and large-scale functional brain networks. We explicitly focused on investigating the characteristics of the brain networks that facilitate perception of the McGurk illusion. We exploited the perceptual variability of McGurk stimuli by comparing the oscillatory responses and network characteristics within identical trials. The main findings of the study are: (1) heightened global coherence in the gamma band along with decreased global coherence in the alpha and theta bands facilitates multisensory perception (2) a broadband enhancement in the global coherence at theta, alpha, beta, and gamma bands aids multisensory perception for asynchronous AV stimuli, as brain engages more energy for multisensory integration. We discuss the behavioral and neural-level findings in following sub-sections.

### Variability of perceptual experience

A vast body of literature has reported that under controlled settings one can induce illusory perceptual experience in human participants (McGurk and Macdonald, 1976; MacDonald and McGurk, 1978; van Wassenhove et al., 2007; Nath and Beauchamp, 2011; Keil et al., 2012). Here, we constructed incongruent AV stimuli (auditory /*pa*/ superimposed onto video of face articulating /*ka*/) using three different AV lags: −450 ms (audio precede articulatory movements), 0 ms (synchronous onsets of audio and articulatory movements), and +450 ms (articulatory movements precede audio) (see Figure 1). We identified that a categorical perceptual difference appeared with variation in AV lags. Synchronous AV stimuli resulted in higher percentage response of crossmodal (/*ta*/) perception (Figure 2C) whereas AV lags of −450 and +450 ms resulted in lowering of the percentage of crossmodal percept and higher occurrence of the unimodal percept /*pa*/. Furthermore, we observed high hit rate of /*ta*/ responses both during congruent /*ta*/ stimuli (>90%) and during our *post-hoc* “auditory alone” behavioral experiment (>95%). Behavioral studies by van Wassenhove et al. (2007) demonstrate 200 ms of asynchrony as the temporal window of bimodal integration. However, electrophysiological studies especially in the domain of preparatory processes demonstrate the elicitation of ERP components up to 600–800 ms in response to a cue followed by a target stimulus (Simson et al., 1977). Extending this line of reasoning to our experimental paradigm, we believe an existence of temporal integration mechanisms beyond 200 ms does not allow the percentage of /pa/ perception to reach the level for congruent multisensory or purely auditory perception. In the current study we focused on the boundaries of stable illusory perception but the temporal boundaries of multisensory integration needs to be tested by future studies.

Interestingly percentage of gaze fixation at the mouth of the speaker for crossmodal response trials did not vary significantly at any AV lags based on *t*-test. Also, the interaction between lags and perceptual categorization was not significant when analyzed with 2-way ANOVA. Even though not statistically significant, the mean gaze fixation percentages at mouth for crossmodal perception were slightly higher than unimodal perception at all AV lags. Therefore, we cannot completely rule out the findings of an earlier study that show that frequent perceivers of McGurk effect fixate more at the mouth of the speaker (Gurler et al., 2015) as well as we were limited by the number of participants to evaluate correlations between the behavioral results and the percentage of gaze fixation at 0 ms AV lag. On the other hand the subjective behavioral response for perceptual categorization clearly showed an interaction effect between AV lags and perceived objects. It is important to note that the identical multisensory stimuli generated varying responses for different trials. All stimuli being multisensory, differential perception served as an efficient handle to tap into the perceptual processing underlying speech perception. Our behavioral response results are consistent with previous studies on McGurk stimuli (Munhall et al., 1996; van Wassenhove et al., 2007) that demonstrate the influence of AV lags on perceptual experience. Hence, we expected to identify the neurophysiological processes underlying different multisensory perceptual scenarios.

### Spectral landscape of the cortical activity

Non-parametric statistical comparison between the perceptual categories (/*ta*/–/*pa*/) showed suppression of the spectral power in alpha, beta, and gamma frequency bands (see Figure 4). Suppression of alpha-band power has been associated with attention and language comprehension processes by enabling controlled access to knowledge (Bastiaansen and Hagoort, 2006; Hanslmayr et al., 2011; Klimesch, 2012; Payne et al., 2013). Accordingly, the suppression of alpha-band power observed in our study can be attributed to the attention related network aiding access to stored knowledge and filter redundant information.

Beta-band power was observed to be suppressed at frontoparietal to occipital sensors during −450 ms AV lag and at occipital scalp regions during 0 ms AV lag but no such suppression was observed during +450 ms AV lag. Beta band power has been linked with various cognitive facets including top-down control of attention and cognitive processing (Engel and Fries, 2010). Besides, in the domain of multisensory integration and language processing, suppression of beta-band power has been associated with the occurrence of unexpected stimuli (Bastiaansen and Hagoort, 2006; Weiss and Mueller, 2012). Furthermore, recent studies also show suppression of beta power during the perception of the McGurk illusion (Roa Romero et al., 2015). Extending the line of reasoning from the aforementioned studies, suppression of beta-band power might be associated to the occurrence of an unexpected stimulus and its processing. Visual-lead condition, wherein we observed no significant difference in the beta power, is possibly the most predictable situation and hence significant beta power modulation was not detected. Behaviorally, Munhall et al. (1996), report McGurk illusion is most dominant between an AV lag of 0–200 ms and there is a slight asymmetry toward positive AV lags (visual lead). In fact, our data from a different experiment also replicated this result.

Gamma-band power was observed to be significantly suppressed only during −450 ms AV lag at the occipital scalp regions. Also, in the pre-stimulus period significant reduction in gamma band power was observed at occipital scalp regions during 0 ms AV lag. Existing studies have demonstrated the role of gamma-band oscillations in cognitive functions like visual perception, attention and in the processing of auditory spatial and pattern information (Jochen Kaiser and Lutzenberger, 2005a,b). Also, gamma band activity over sensory areas has been attributed to the detection of changes in AV speech (Kaiser et al., 2006). However, we observed a suppression in gamma band activity which may be linked with preparatory processes over wider network that waits for the expected visual information to arrive. Although, the brain oscillatory responses to multisensory perception have been extensively studied, a consensus on the mechanisms associated with these oscillations remains elusive. Our study contributes to this vast body of work in conveying that multisensory speech perception requires complex signal processing mechanisms that involves the participation of several brain regions. Therefore, understanding the process requires analyzing the whole brain operating as large scale neurocognitive network. In the subsequent section we discuss the network analysis results.

### Neurocognitive-network level processing underlying illusory perception

Global time-frequency coherogram (see Figure 5) computed for the perceptual categories quantifies the extent of coordinated neuronal activity over the whole brain. Global coherence reflects the presence of neuro-cognitive networks in physiological signals (Bressler, 1995). Previous studies posits that neuronal coherence could provide a label that binds those neuronal assemblies that represent same perceptual object (von der Malsburg and Schneider, 1986; Engel, 1997; Engel et al., 2001). Besides, going by the communication-through-coherence (CTC) hypothesis, only coherently oscillating neuronal groups communicate effectively as their communication window for spike output and synaptic input are open at the same time (Senkowski et al., 2008; Fries, 2015). Hence, coherent transmission poses a flexible mechanism that facilitates the integration of converging streams in time windows of varying duration. In our analysis we observed a relatively heightened theta-band coherence for both the perceptual categories at all the AV lags (see Figures 5A,B,D,E,G,H). Theta band coherence has been associated to cognitive control processes (Cooper et al., 2015). Accordingly, the enhanced theta-band coherence might reflect the control processes preparing for upcoming stimuli.

Non-parametric statistical analysis employed to test the global coherence differences between /*ta*/ and /*pa*/ during 0 ms AV lag, revealed a positive cluster, signifying enhanced synchrony specifically at the gamma band (between ~300 ms and 700 ms). Also, we observed negative clusters (between ~300 and 900 ms) in the theta, alpha and beta bands that signify decreased synchrony among the underlying brain regions. Overall temporal congruence of AV stimuli results in a narrow-band coherence whereas lagged AV stimuli seemed to engage a broadband coherence (see Figure 5C,F,I). However, we had one limitation because of the nature of our stimuli. A direct statistical comparison across lagged conditions was not meaningful since each lagged condition had a different temporal sequence of audio-visual components.

Inter-areal coherence of oscillatory activity in the beta frequency range (15–30 Hz) has been associated with top-down processing (Wang, 2010). Moreover, top-down processing involves the modulation of the hierarchical sensory and motor systems by pre-frontal and frontal brain areas (Mesulam, 1990). The dense anatomical interconnectivity among these association areas give rise to self-organized large scale neuronal assemblies defined as neuro-cognitive networks (NCNs), with respect to the cognitive demands (Bressler and Richter, 2014). In this context, our finding of increased coherence in the beta band during −450 and +450 ms AV lag is especially relevant as it enables us to hypothesize that synchronization of the beta oscillations provides long range inter-areal linkage of distributed cortical areas in NCNs. Such networks can readily process the retrieval of well learnt audio-visual associations suggested by Albright (2012).

Gamma band coherence are shown to be associated with voluntary eye movements, saccades (Balazs et al., 2015). Besides, stimulus selection by attention also induces local gamma band synchronization (Hipp et al., 2011). Our results show enhanced gamma coherence (positive cluster) at all AV lags. Considering the increased gaze fixation at mouth during /*ta*/ perception, heightened gamma coherence reflects the recruitment of the visual attention areas. A recent review proposes that gamma band (30–90 Hz) coherence activates postsynaptic neurons effectively by modulating the excitation such that it escapes the following inhibition (Fries, 2015). Besides rendering effective communication, gamma coherence has also been proposed to render communication that are precise and selective (Buzsáki and Schomburg, 2015; Fries, 2015). Importantly, gamma band coherence has also been demonstrated to be implicated in associative learning (Miltner et al., 1999). Thus, our observation of enhanced coherence exclusively at gamma and desynchronization at alpha and beta-bands during 0 ms AV lag portrays an attention network working in harmony with the NCNs most likely linked to associative memory retrieval. This conjecture is also supported by the secondary evidence in case of −450 and 450 ms AV lags, where an additional working memory process is competing for processing and integration of the multisensory stimuli and leading to a broadband enhancement in global coherence. A more detailed delineation of working memory processing and associative memory recall needs to be carried out with other kinds of multisensory stimuli and will be a major focus of our future endeavors.

## Author contributions

GK and AB designed the study; GK, AM, and AB designed the stimulus; GK and AJ recorded the data; GK, TH, DR, and AB analyzed the data; GK, DR, and AB wrote the manuscript; GK, TH, AJ, AM, DR, and AB read and commented on the manuscript.

### Conflict of interest statement

The authors declare that the research was conducted in the absence of any commercial or financial relationships that could be construed as a potential conflict of interest.
